# Cohort research analysis of disaster experience, preparedness, and competency-based training among nurses

**DOI:** 10.1371/journal.pone.0244488

**Published:** 2021-01-08

**Authors:** Krzysztof Goniewicz, Mariusz Goniewicz, Frederick M. Burkle, Amir Khorram-Manesh

**Affiliations:** 1 Department of Aviation Security, Military University of Aviation, Dęblin, Poland; 2 Department of Emergency Medicine, Medical University of Lublin, Lublin, Poland; 3 Harvard Humanitarian Initiative, T.H. Chan School of Public Health, Harvard University, Boston, MA, United States of America; 4 Department of Surgery, Sahlgrenska Academy, Institute of Clinical Sciences, Gothenburg University, Gothenburg, Sweden; 5 Department of Development and Research, Armed Forces Center for Defense Medicine, Gothenburg, Västra Frölunda, Sweden; Technion - Israel Institute of Technology, ISRAEL

## Abstract

**Introduction:**

It is expected that in unforeseen situations, nurses will provide appropriate medical interventions, using their expertise and skills to reduce the risks associated with the consequences of disasters. Consequently, it is crucial that they are properly prepared to respond to such difficult circumstances. This study aimed to identify the factors influencing the basic competences of nurses in disasters.

**Materials and methods:**

The survey was directed to 468 nurses from all medical centres in Lublin. IBM SPSS Statistics version 23 was used for statistical analyses, frequency analysis, basic descriptive statistics and logistic regression analysis. The classical statistical significance level was adopted as α = 0.05.

**Results:**

Based on the logistic regression analysis, it was found that work experience, workplace preparedness, as well as training and experience in disaster response are important predictors of preparedness.

**Conclusions:**

These findings indicate that the nurses' core competencies for these incidents can be improved through education and training programmes which increase their preparedness for disasters. Nurses are among the most important groups of healthcare professionals facing a disaster and should be involved in all phases of disaster management, such as risk assessment and pre-disaster planning, response during crisis situations and risks’ mitigation throughout the reconstruction period.

## 1. Introduction

In today's world, nursing is becoming an increasingly important medical profession, striving to provide people with ever better support in achieving optimal health. Currently in Poland nurses are full partners in the therapeutic team, and are entitled to propose solutions in a patient's therapeutic process and perform medical interventions. Thus, they are responsible for the decisions taken with regard to the patient and are obliged to act in accordance with current medical knowledge and practice, the code of professional ethics and applicable legal standards. A high level of education and increased professional competence havs resulted in new functions and tasks for nurses, e.g. responding to disasters. Nurses play a key role in emergency preparedness at a local, state and national level through planning, social and consumer education and the direct care provided during disasters [1].

The World Health Organisation defines a disaster as 'an occurrence disrupting the normal conditions of existence and causing a level of suffering which exceeds the capacity of adjustment of the affected community' [2]. From a healthcare point of view, a disaster is an event where the number of victims and medical needs exceed the capabilities and capacities of the existing healthcare system.

In most countries, nurses constitute the largest group of healthcare workers who are actively involved in caring disaster victims. However, they also offer their expertise to the community of affected people, staying with them for a substantial period of time after the event. It is, thus, crucial that nurses are properly prepared for such difficult situations. In particular, nurses working in hospitals, e.g. emergency, intensive care and trauma wards, must have the necessary knowledge, skills and experience to effectively manage crisis situations and the often-chaotic circumstances accompanying disasters [3]. The main objective of rescue operations in disasters is to save the life of people in conditions exceeding the capabilities of everyday medical care. Before any disaster occurs, one priority should be nurses’ education, enabling them to provide an effective response and help to a large number of victims using limited resources during a mass casualty incident [4].

While other studies, as well as one recent study in Lublin, have measured aspects of preparedness among physicians, there are few reports globally which directly measure aspects of nurses’ preparedness in responding to disasters [5–7]. To date, there has not been any research conducted in Poland regarding the analysis of nurses' preparedness for disasters. This study has been designed to better understand the opinions and insights of nurses working in Lublin concerning their role, knowledge, and experience with regard to disaster response. Understanding the competencies of nurses can be used to develop strategies for their effective use during disasters.

## 2. Materials and methods

### 2.1 Study location

This study was carried on in May and June 2020 in the city of Lublin, which is the ninth largest city in Poland in terms of population, and sixteenth in terms of area. Lublin is the capital of the Lubelskie Province and has one of the most important and flourishing academic hubs in Poland, including primary and specialist healthcare centers, several clinical hospitals (University Hospitals, Military Clinical Hospital, Provincial Specialist Hospital, Neuropsychiatric Hospital, and the Ministry Hospital). It also has an oncology center and an institute for Rural Medicine, and thus, a significant role in educating future medical staff in the region.

### 2.2 Study population

The survey was directed to 468 nurses from all medical centres in Lublin. Since many healthcare providers may work in several places, the participants were asked to report a primary workplace. There was also no subdivision between acute and critical care nurses, which will be included in further studies, as it is obvious that the variables studied will differ significantly between both groups.

### 2.3 Questionnaire

In the first step of this study, all authors took part in a literature review to identify the critical dimensions for developing a questionnaire. For the review purpose, the following keywords: disaster nurses; disaster nursing education; emergency preparedness for nurses; nurses' disaster preparedness, nurses training. alone or in combination were used. The acquired data from PUBMED, SCOPUS, and WEB OF SCIENCE were organized, categorized, and mapped.

The following dimensions of preparedness: perception of disaster risk, the experience of disaster response, disaster training, and preparedness for particular threats for disasters that are likely to be reported in the regional crisis management plans were assessed. We have only investigate preparedness for disasters that are likely to be reported in the regional crisis management plans. Therefore, when creating the survey, we also used such keywords (in Polish) as: Lublin threats, Lublin disasters; Lublin emergency plans.

It took 5–10 minutes to complete the questionnaire (S1 Appendix), which consisted of 13 questions distributed in all four domains: the perceived preparedness; the experience of disaster response; the remaining questions were demographic and covered the rest of the dimensions.

In the second step, a group of 15 employees from one university hospital was used to evaluate the questionnaire. These participants were, then, excluded from the study, and their responses were not used in the final analysis. The outcome was reviewed based on a combination of logic, relevance, comprehension, legibility, clarity, and usability before the final administration.

The questionnaire used in this research is available online. All relevant data in Polish are within the manuscript and its supporting information files (S2 Appendix).

### 2.4 Data collection

The questionnaire was presented in both written and digital versions due to the current COVID-19 pandemic. Four hundred and sixty-eight nurses were provided with the survey, and all completed it.

### 2.5 Statistical analysis

IBM SPSS Statistics version 23 was used for statistical analyses, frequency analysis, basic descriptive statistics and logistic regression analysis. The classical statistical significance level was adopted as α = 0.05.

### 2.6 Ethical considerations

The information included the study’s purpose, the voluntary nature of their participation, and strict confidentiality and secure data storage. The survey had anonymous nature and all respondents agreed to participate in the survey. Verbal consent was obtained from participants who completed the paper questionnaire and written from those who completed the online questionnaire. It complied with the ethical principles stipulated by Polish law and thus was exempted from ethics approval requirements. Under this provision, approval from the IRB was not needed as the study is not a medical experiment and legally does not require the opinion of the Bioethics Committee within the meaning of Polish Law and according to the Act of 5 December 1996, the professions of doctor and dentist. The Polish Sejm site: https://isap.sejm.gov.pl/isap.nsf/DocDetails.xsp?id=WDU19970280152 (in Polish).

## 3. Results

The majority of respondents were women (88.5%). The most numerous age group consisted of respondents from 35 to 44 years old (34.8%). As a primary workplace, the respondents mainly chose the public hospital (79.9%). The largest group of respondents were nurses with over 20 years of experience (22.2%). The results concerning demographic data are presented in Table 1.

**Table 1:** Demographic data.

Age	*N*	%
Up to 34	134	28.6%
35–44 years	163	34.8%
45–54 years	122	26.1%
55 years and over	49	10.5%
**Gender**	*N*	%
Female	414	88.5%
Male	54	11.5%
**Length of service**	*N*	%
From 0 to 5 years	92	19.7%
6–10 years	71	15.2%
11–15 years	100	21.4%
16–20 years	101	21.6%
More than 20 years	104	22.2%
**Workplace**	*N*	%
Public hospital	374	79.9%
Research facility	94	20.1%

In the first part of the survey, the respondents were asked about the likelihood of various disasters occurring in the city of Lublin within the next 5 years. The majority of nurses believed that the occurrence of floods is likely (38.9%) or possible (27.6%). In the event of an epidemic or pandemic, the majority of the surveyed group indicated that the occurrence of such an event is likely (40.6%). More than a quarter of the surveyed group indicated that the risk of epidemic occurrence was very high (28.2%) or possible (25.6%). When asked about a terrorist or bioterrorist attack, most respondents indicated a low risk (41.7%) of such an event occurring. Moreover, 23.5% of the respondents described the risk of such an event as very low. However, a quarter of the respondents (25%) considered its occurrence to be possible. In the case of a chemical disaster, the most numerous group were the respondents who believed that there is a low risk (40.8%) of such an event occurring, while 34.4% indicated that such a disaster is likely to occur. As far as a plane crash is concerned, 37% of the respondents indicated that the occurrence of such an event is possible, 34% said that there is a low risk of such an event, and 21.2% assessed the risk as very low. Concerning a railway crash, 44.2% of nurses indicated that it is possible, 34.4% of respondents said that there is a low risk of such a crash and 8.8% described this risk as very low. In the case of drought, 36.5% of the respondents considered it probable and 33.3% considered it possible. Very high risk of drought was indicated by 7.9% and very low risk by 2.8% of respondents. Most of the respondents (41.9%) described the likelihood of a large fire in Lublin as possible. Whereas 4.1% assessed this risk as very low. Almost half of the nurses surveyed (49.6%) indicated a very low risk of this event, and 32.1% rated it as low. The results of answers to questions concerning the risk of disasters in Lublin are presented in Table 2.

**Table 2:** Likelihood of disasters occurring in Lublin in the next 5 years.

Risk of disaster occurring	Very low		Low		Possible		Probable		Very High	
Type of incident	*N*	%	*N*	%	*N*	%	*N*	%	*N*	%
Flooding	32	6.8%	56	12.0%	129	27.6%	182	38.9%	69	14.7%
Epidemic	0	0.0%	26	5.6%	120	25.6%	190	40.6%	132	28.2%
Terrorist/bioterrorist attack	110	23.5%	195	41.7%	117	25.0%	38	8.1%	8	1.7%
Chemical disaster	63	13.5%	191	40.8%	161	34.4%	40	8.5%	13	2.8%
Air crash	99	21.2%	159	34.0%	173	37.0%	31	6.6%	6	1.3%
Railway crash	41	8.8%	161	34.4%	207	44.2%	53	11.3%	6	1.3%
Drought	13	2.8%	91	19.4%	156	33.3%	171	36.5%	37	7.9%
Large fire	19	4.1%	109	23.3%	196	41.9%	134	28.6%	10	2.1%
Earthquake	232	49.6%	150	32.1%	72	15.4%	14	3.0%	0	0.0%

In the following part of the study, nurses were asked whether they had provided assistance to victims of disasters in Lublin or elsewhere. Most of the respondents had helped victims in two types of disasters, i.e. floods and epidemic. Out of the respondents who had helped flood victims, over 60.5% had helped in a location other than Lublin. Around 95.6% of investigated nurses had helped victims of epidemics in Lublin. In the case of other disasters, most respondents had helped victims in a location other than Lublin. The results are presented in Table 3.

**Table 3:** Experience in helping disaster victims.

Type of incident	Place of incident: Lublin		Other	
	*N*	%	N	%
Flooding	51	39.5%	78	60.5%
Epidemic	263	95.6%	12	4.4%
Terrorist/bioterrorist attack	3	60.0%	2	40.0%
Chemical disaster	4	66.7%	2	33.3%
Air crash	0	0.0%	3	100.0%
Railway crash	0	0.0%	13	100.0%
Drought	0	0.0%	8	100.0%
Large fire	8	42.1%	11	57.9%
Earthquake	0	0.0%	3	100.0%

The participants were then asked about their training. Most of the respondents (97.4%) had received first aid training, and more than half of the respondents (67.7%) had received BLS (Basic Life Support training. The opposite was true for ALS (Advanced Life Support) and ACLS (Advanced Cardiovascular Life Support) training, i.e.: 65.4% of the respondents had not participated in ALS training and 88.7% had not received ACLS training. Similarly, the majority of the respondents reported that they had not participated in any of the following training: triage (83.1%), psychological care (92.7%), crisis management (94%), humanitarian law (97.4) and hazardous materials (HAZMAT) (95.9%). The results are presented in Table 4.

**Table 4:** Training courses completed.

Type of training	Yes		NO	
	*N*	%	N	%
First aid	456	97.4%	12	2.6%
BLS	317	67.7%	151	32.3%
ALS	162	34.6%	306	65.4%
ACLS	53	11.3%	415	88.7%
Triage	79	16.1%	389	83.1%
Psychological care	34	7.3%	434	92.7%
Crisis management	28	6.0%	440	94.0%
Humanitarian law	12	2.6%	456	97.4%
HAZMAT	19	4.1%	449	95.9%

**Fig 1:**
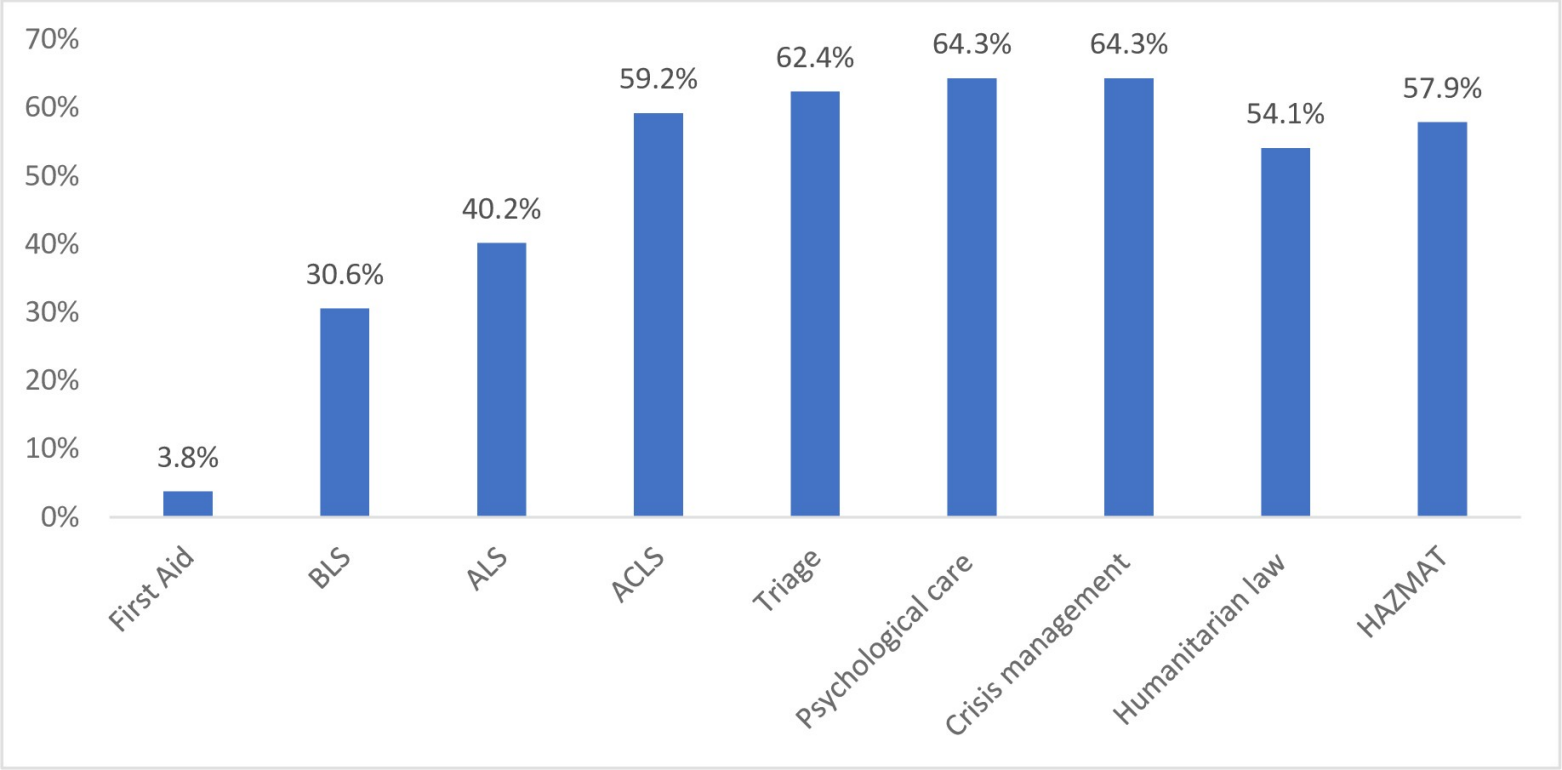
Preferences for specific training.

Next, the respondents were asked to mark the training courses they would like to have. The most frequently selected training courses included: crisis management (64.3%), psychological care (64.3%) and triage (62.4%). The results are presented in the Fig 1.

When asked whether their workplace offers disaster preparedness training, the majority (89.1%) of nurses surveyed responded negatively. Only 10.9% of respondents reported the existence of such an opportunity at their workplace.

In the following part of the survey, basic descriptive statistics were calculated for variables measured quantitatively. In the case of assessing the level of risk of disaster occurrence in Lublin within the next 5 years, the average level of this variable and the median value is 3.00, with a standard deviation of 0.95. The lowest value in the distribution is 1 and the highest is 5.

The average level of self-assessment of one's own preparedness for a disaster in the examined group is 2.32, deviating +/- by 0.87. The median value of this variable is 2.00. The lowest value obtained is 1 and the highest is 5.

The mean value of the assessment of workplace preparedness for a disaster is 2.70, with a standard deviation of 0.82. The mean value is 3.00. The range of this variable is 4, from 1.00 to 4.00.

**Fig 2:**
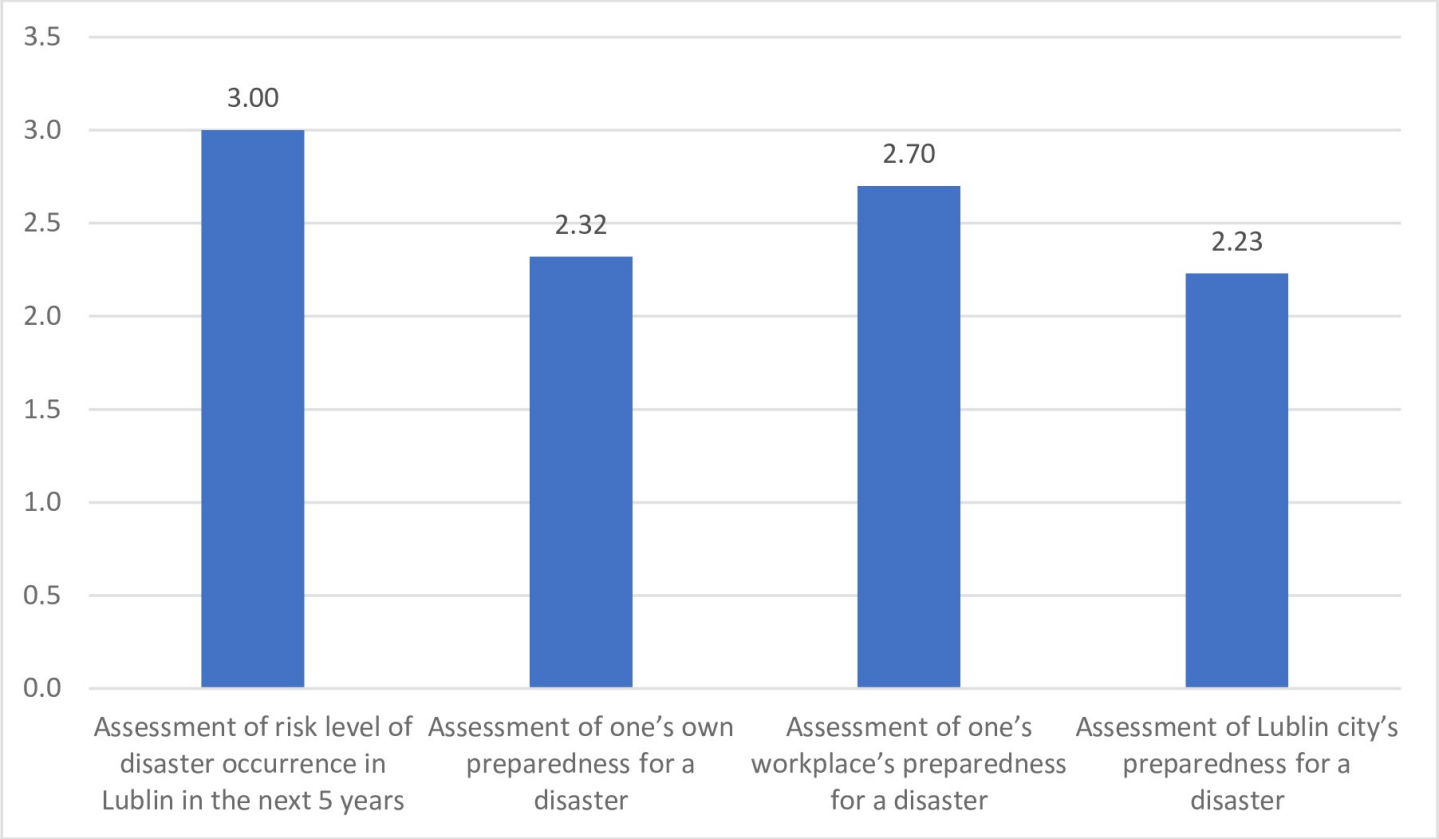
Averages for the assessment of risk level of disaster occurrence and preparedness for disasters. Y axis = Likert scale from 0–5.

As regards the assessment of Lublin city's disaster preparedness, the average level of the variable is 2.70, with a deviation of +/- by 0.82. The median value for this variable is 3.00. The lowest value recorded in this distribution is 1.00 and the highest is 4.00. The results are presented in Fig 2.

In the last step of the statistical analysis, logistic regression analysis was performed using the backward selection technique. It was decided to test whether the predictors (length of service, workplace preparedness, experience in disaster response and training) foresee an assessment of one’s own preparedness for disasters.

The last model is properly matched to the data: χ2(6) = 127.10; p < 0.001. This model explains 64% of the variance of the dependent variable (R2 Nagelkerke = 0.64) and fits 96.4% of the cases accordingly. Its specificity is 99.3%, while its sensitivity is 51.7%.

Length of service, workplace preparedness, triage training are important predictors. In cases of more than 15 years length of service, the assessment of one's own preparedness as good increases by 7.31 times. Moreover, if the respondents assess the preparedness of the workplace as good, the chance of assessing one's own preparedness as good increases by 23.1 times. In addition, those who have undergone triage training have a 73.41-times higher chance of assessing their own preparedness as good.

The values of the logistic regression analysis coefficients for the assessment of one's own preparedness are presented in Table 5.

**Table 5:** Regression coefficients foreseeing good self-preparedness for disasters.

	*B*	*SE (B)*	Wald	*p*	Exp(*B*)	95% *CI*	
						LL	UL
Constant	-2,29	1,01	5,19	0,023	0,10		
Length of service	1,99	0,69	8,31	0,004	7,31	1,89	28,28
Workplace preparedness	3,14	0,69	20,50	<0,001	23,10	5,93	89,92
Training in triage	4,30	0,73	34,82	<0,001	73,41	17,62	305,86

B–unstandardised regression coefficient; SE–standard error; Wald–test to determine the statistical significance of predictors; p–Significance; Exp(B)–odds ratio; 95% CI– 95% confidence interval with lower limit (LL) and upper limit (UL)

## 4. Discussion

This study assessed the level of preparedness of nurses for disasters and shed some light on a better understanding of the role and responsibilities to be fulfilled by nurses in Lublin and Poland in response to a disaster. Despite significant national investment in the development of new programmes for disaster prevention, the preparation of communication infrastructure, and the dissemination of knowledge in this field, the respondents who participated in this survey reported a low level of knowledge in effective disaster response. Also worrying is the lack of knowledge in medical preparation, especially in regards to advanced resuscitation activities (ALS, ACLS), as well as the low self-assessment of nurses concerning preparedness for disasters. A recent study using the questions from this survey was conducted among physicians in Lublin with different outcomes. Consequently, and due to differences in nurses’ and physicians’ educational backgrounds and disaster management practices, the results in this study are presented separately [20].

Disaster mitigation focuses on preparation, prevention, response and reconstruction. Disaster response plans should be available before a disaster occurs. Additional resources and training are needed to cope with the particular challenges posed by disasters. In most countries, nurses are the largest group of health professionals, actively involved with disaster victims [6]. The accumulated literature suggest several factors, which may influence the ability of nurses to respond to a disaster [7–9]. Disaster education and training is one key element. Exercises, as well as training and post-graduate studies focusing on disaster response, are important aspects of education and training activities [10]. Nevertheless, there is less consensus in the literature on the content and manner of implementation of such educational programmes. The second factor influencing the effectiveness of nurses' disaster response is their level of knowledge and awareness of the right course of action. The third key element is experience [11].

Most of the respondents, in this study, had not received the training needed for proper response to a disaster, particularly courses in triage and psychological care were missing. It is crucial that nurses are properly prepared to act in difficult circumstances caused by unforeseen situations. It is worrying that almost 96% of nurses, in this study, have not received HAZMAT training, which in the era of the COVID-19 pandemic would seem to create additional problems. The nurses who had undergone training in triage, psychological care, and HAZMAT evaluated their own preparedness significantly better than those who had not been involved in such training. This study also revealed that nurses seem reluctant to play a leading role in disaster response or the provision of prophylactic and psychological care. However, these are essential skills for any nurse who may be called upon to make decisions or support patients affected by stress, panic or other emotional responses during a disaster. It is also worth noting that those of the respondents who had prior experience in disaster response are better prepared for such events. The number of courses held also significantly increases preparedness for mass casualty incidents. Nurses should actively participate in all phases of preparation and response to disasters in their institutions and communities [12, 13].

The level of preparedness of nurses is a key element in terms of their disaster response. In our sample, education and training in disaster preparedness vary greatly depending on the length of time that nurses have been working. Participation in disaster-related exercises must become more standardised and include all nurses [14]. Training programs need to be changed to better prepare them for mass casualty incidents. Such training should include prevention, monitoring, overall patient assessment and care, triage, resuscitation, and psychological care. The selection of teachers—their qualifications, including a comprehensive understanding of the specificity of crisis situations and assistance needed at that time, their experience, knowledge and cultural sensitivity—is also crucial. Often, just after a disaster, many training sessions are conducted to quickly supplement the skills of personnel. Nurses are then at risk of information overload, which is why as much training as possible should be devoted to developing the knowledge and skills which they already possess. The introduction of new concepts and competences should be limited to the absolute minimum [15, 16]. From the nurses' answers to the questionnaire, it appears that at present the nurses' pre and post-graduate education places less emphasis on areas such as crisis management, disaster medicine and psychological care. Training is directed towards clinical tasks related to the provision of emergency care, rather than more proactive clinical management and prevention activities [17–20].

The experience of the nurses from particular disasters has so far been related to the COVID-19 pandemic and floods. For this reason, it is difficult to say how such a reaction would actually look in the event of other incidents. For example, a study in New Zealand showed that despite a low self-reported preparedness among acute care providers, the healthcare service was found to have “responded well to extraordinary circumstances” in the Canterbury earthquakes in 2010 and 2011 [21]. On the other hand, numerous studies show that healthcare workers, confident of their own high competences, are more likely to react effectively in real crises than workers who perceive their competences as low [22–27].

Although the results of this study concern one specific area in a country, it can be generalize to other areas and countries worldwide. The current COVID-19 pandemic is a fresh example of how a local threat can become a world issue [28]. Local and regional preparedness is thus crucial for the global well-being [29]. Further research is needed to investigate the relationship between nurses' perceived preparedness and their actual response to disasters. Additionally, these findings can be used as crucial and measurable parameters in future tabletop or simulation exercises.

## 5. Limitations

The main limitation of this study was that it examined only a limited number of nurses from the city of Lublin. The COVID-19 pandemic proved to be an obstacle to further research. There was also no subdivision between acute and critical care nurses, which will be included in further studies, as it is obvious that the variables studied will differ significantly between both groups. Despite these limitations, the study revealed gaps and training needs in terms of preparing nurses for disasters. It also opens up a discussion on this subject and the perspective of broader research in this area. Having accumulated valuable experience from a large number of responders, the survey gains mandate to be used for examination of a larger and diverse group of healthcare professionals and enables the authors to form the basis for planned future research. During the nationalization of the survey, its content will be discussed and modified, if necessary, to serve as a wider standardisation of the research tool.

The online questionnaire was sent to the Medical University staff mailboxes and the paper version to all nurses at the medical university. However, only 486 nurses responded. Since we are unable to determine how many people really received the survey. Thus, we cannot estimate the response rate. Nevertheless, this is the first study on the subject of nurses' preparedness for disasters in Poland, and one of the few studies in Europe which provides valuable and new information on nurses' perceived preparedness for mass casualty incidents, and indicates a need for further in-depth research in this area.

Finally, earlier studies have shown that the knowledge of own workplace preparedness in responding to disasters, increases the willingness to work during a disaster and acquire the knowledge necessary to respond [30]. The fact that our respondents had to choose their working place due to having several workplaces might be another limitation. However, since only 10% received training, the impact should not be so high.

## 6. Conclusions

Nurses are among the most important groups of healthcare professionals facing a disaster and should be involved in all phases of disaster management, such as risk assessment and pre-disaster planning, response during crises and risks’ mitigation throughout the reconstruction period. Consequently, they should be prepared by educational initiatives and training. The aim of an education initiative for nurses is to acquire the knowledge, skills and competences necessary to provide rapid and specialized treatment and care in situations where life is suddenly threatened, in accordance with applicable standards of conduct, as well as preparing nurses to perform professional nursing duties in the emergency medical system [31]. Responsibility for the training of nurses must be overseen by decision-makers at the local and national level.

## Supporting information

S1 Appendix Questionnaire. (DOCX)

S2 Appendix. Data Statistical data, database, results. (XLSX)
