# Supplementary material for: Cohort research analysis of disaster experience, preparedness, and competency-based training among nurses
Source: PLoS One. 2021 Jan 8;16(1):e0244488. doi: 10.1371/journal.pone.0244488 (PMC7793243; doi:10.1371/journal.pone.0244488)
Supplement: S1 Appendix — (DOCX) [file pone.0244488.s001.docx]

**S1 Appendix**

**Questionnaire: "Preparedness of nurses in the event of disasters"**

*Please read the questionnaire carefully and fill it in. When answering, please tick the appropriate boxes. The survey is completely anonymous. Your responses will only be used for statistical summaries.*

1. **How do you assess the general level of risk of an occurrence in Lublin within the next 5 years, on a scale of 1 to 5?**

*Very low risk* 1 2 3 4 5 *Very high risk*

1. **How do you assess the likelihood of an event in Lublin within the next 5 years, on a scale of 1 to 5?**

*(Where 1 is very low and 5 very high))*

Flooding 1 2 3 4 5

Epidemic 1 2 3 4 5

Terroris / bioterrorist attack 1 2 3 4 5

Chemical disaster 1 2 3 4 5

Air Crash 1 2 3 4 5

Railway crash 1 2 3 4 5

Drought 1 2 3 4 5

Large Fire 1 2 3 4 5

Earthquake 1 2 3 4 5

1. **Have you helped victims of any of the following events, in Lublin or other place?**

Lublin Other Place

Flooding
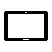

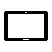


Epidemic
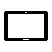

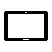


Terrorist/bioterrorist attack
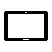

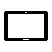


Chemical disaster
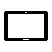

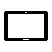


Air crash
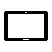

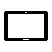


Railway crash
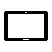

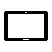


Drought
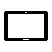

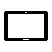


Large fire
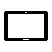

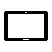


Earthquake
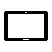

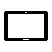


1. **Have you received any of the following trainings?**

YES NO

First Aid
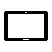

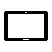


ALS
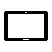

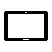


BLS
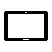

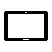


ACLS
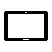

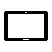


Triage
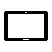

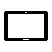


Psychological care
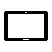

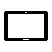


Crisis management
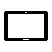

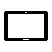


Humanitarian law
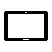

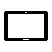


HAZMAT/CBRN
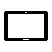

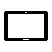


1. **Which training would you like to participate?**
2. First AID
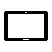

3. ALS
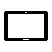

4. BLS
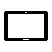

5. ACLS
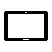

6. Triage
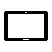

7. Psychological care
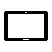

8. Crisis management
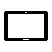

9. Humanitarian law
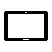

10. HAZMAT/CBRN
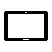

11. **Does the workplace offer trainings / exercises related to preparation for disaster?**

- Yes
- No

1. **How do you assess your own preparation for a disaster, on a scale of 1 to 5?**

Very low 1 2 3 4 5 very well

1. **How do you assess the current level of preparation of your workplace in case of a disaster, on a scale of 1 to 5?**

Very low 1 2 3 4 5 very well

1. **How do you assess the current level of preparation for the accident of the city of Lublin, on a scale of 1 to 5?**

Very low 1 2 3 4 5 Very well

1. **Gender:**

- Men
- Women

1. **Age:**

- Up to 34
- 35-44
- 45-54
- 55 years and over

1. **Workplace**

- Public hospital
- Research facility
- Outpatient Clinic

1. **Length of service:**

- From 0 to 5 years
- 6-10
- 11-15
- 16-20
- More than 20 years

*Thank you for completing the survey*
